# Oral Insulin Alleviates Liver Fibrosis and Reduces Liver Steatosis in Patients With Metabolic Dysfunction-associated Steatohepatitis and Type 2 Diabetes: Results of Phase II Randomized, Placebo-controlled Feasibility Clinical Trial

**DOI:** 10.1016/j.gastha.2023.11.016

**Published:** 2023-12-07

**Authors:** Yuval Ishay, Joel Neutel, Yotam Kolben, Ram Gelman, Orly Sneh Arbib, Oliver Lopez, Helena Katchman, Rizwana Mohseni, Miriam Kidron, Yaron Ilan

**Affiliations:** 1 Department of Medicine, Hadassah Medical Center and Faculty of Medicine Hebrew University, Jerusalem, Israel; 2 Orange County Research Center, Tustin, California; 3 Integrium LLC, Tustin, California; 4 Sourasky Medical Center, Tel Aviv, Israel; 5 Catalina Research Institute, Montclair, California; 6 Oramed Pharmaceuticals, Israel

**Keywords:** NAFLD, MASH, Insulin, Oral, Diabetes Type 2

## Abstract

**Background and Aims:**

Metabolic dysfunction-associated steatohepatitis is an advanced form of nonalcoholic fatty liver disease and a leading cause of end-stage liver disease and transplantation. Insulin resistance and inflammation underlie the pathogenesis of the disease.

**Methods:**

This double-blind, randomized, placebo-controlled, multicenter feasibility clinical trial aimed to determine the safety of oral 8 mg insulin in patients with metabolic dysfunction-associated steatohepatitis and type 2 diabetes mellitus. Patients were treated twice daily for 12 weeks with an 8 mg insulin (n = 21) or placebo (n = 11) capsule. Safety was monitored throughout the study. MRI-proton density fat fraction assessed liver fat content, and Fibroscan® measured liver fibrosis and steatosis levels at screening and after 12 weeks of treatment.

**Results:**

No severe drug-related adverse events were reported during the study. After 12 weeks of treatment, mean percent reductions in whole-liver (−11.2% vs −6.5%, respectively) and liver segment 3 (−11.7% vs +0.1%, respectively) fat content was higher in the insulin than in the placebo arm. Patients receiving insulin showed a median −1.2 kPa and −21.0 dB/m reduction from baseline fibrosis and steatosis levels, respectively, while placebo-treated patients showed median increases of 0.3 kPa and 13.0 dB/m, respectively. At Week 12, oral insulin was associated with a mean of 0.27% reduction and placebo with a 0.23% increase from baseline hemoglobin A1c levels. Mean percent changes from baseline alanine aminotransferase, and aspartate aminotransferase levels were −10% and −0.8%, respectively, in the oral insulin and 3.0% and 13.4%, in the placebo arm.

**Conclusion:**

The results of this feasibility study support the safety and potential therapeutic effect of orally delivered insulin on liver fibrosis, fat accumulation, and inflammatory processes (NIH Clinical Trials No. NCT04618744).

## Introduction

Nonalcoholic fatty liver disease (NAFLD) comprises liver steatosis, which can progress to metabolic dysfunction-associated steatohepatitis (MASH), cirrhosis, and hepatocellular carcinoma. NAFLD is a significant health burden, and its incidence is increasing worldwide.^1^

Metabolic and inflammatory mechanisms contribute to the development of MASH,^2^^,^^3^ with insulin resistance playing a significant role in the pathogenesis of the disease, as reflected by a reduced hepatic, whole body, and adipose tissue insulin sensitivity in this patient population.^4^^,^^5^ NAFLD and MASH are associated with the excess flow of free fatty acids arising from insulin-resistant adipose tissue, leading to fat deposition in numerous organs.^6^ Fat accumulation in the liver also evolves from excess dietary fat, inadequate fatty acid oxidation, and increased de novo lipogenesis.^7^ When the lipid supply surpasses the liver's metabolic capacity, free fatty acids are redirected toward harmful pathways of nonoxidative metabolism. It leads to intracellular accumulation of toxic lipid-derived metabolites associated with mitochondrial dysfunction, endoplasmic reticulum stress, and activation of inflammatory pathways.^5^^,^^8^ Once NAFLD is diagnosed, liver fibrosis is the central determinant of hepatic prognosis.^9^

Treatment of MASH is a significant challenge. Weight reduction by lifestyle modification or surgery is an effective therapy that is difficult to sustain and can involve invasive procedures.^10^ Available pharmacological and nonpharmacological therapies target different aspects of the pathogenesis of the disease, have limited efficacy, and may be associated with potentially harmful side effects, as decades of treatment are required.^9^^,^^11^, ^12^, ^13^ Current pharmacologic therapy for NAFLD is limited mainly to vitamin E and pioglitazone, while various agents are being investigated in clinical trials.^9^^,^^14^^,^^15^ As multiple mechanisms underlie disease pathogenesis, most currently developed drugs, do not achieve satisfactory results in most patients, as they target a single pathway.^12^

As insulin resistance is a significant pathogenetic factor in MASH16, insulin administration has beneficial effects on liver pathology and expression of genes related to inflammation and fibrosis.^16^ Oral 8 mg insulin (ORMD-0801) was recently shown to be effective in patients with type 2 diabetes mellitus (T2DM) without increasing the risk of hypoglycaemia.^17^ The oral formulation is absorbed through the gut in a biologically active form, lowering serum glucose levels and suppressing endogenous insulin secretion.^18^ It is expected to improve the liver and adipose tissue insulin resistance states, thereby alleviating a significant pathogenetic mechanism of MASH. The present double-blinded, randomized, placebo-controlled study aimed to determine the safety of ORMD-0801 in patients with MASH and T2DM.

## Methods

### Study Design

The study was a double-blinded, randomized (2:1), placebo-controlled, multicenter, exploratory study conducted in 5 medical centers in the US and Israel during 2021-22. The local institutional review boards and ministries of health approved the study. All patients provided signed informed consent before enrollment (NIH Clinical Trials No. NCT04618744).

During the screening period, clinical laboratory, blood lipid, and hemoglobin A1c (HbA1c) evaluations, 12-lead electrocardiogram, a complete physical examination, CAP-FibroScan, and MRI-proton density fat fraction (MRI-PDFF) were performed. After the screening phase, patients participated in a 2-week placebo run-in period, followed by a 12-week treatment phase. Patients were treated twice daily for 12 weeks with an 8 mg ORMD-0801 (n = 21) or placebo (n = 11) soft gel capsule. Capsules were taken in the morning, approximately 30–45 min prior to breakfast, no later than 10 AM, and in the evening, between 8 PM and midnight, and no sooner than 1 hour after dinner. During the run-in and treatment periods, self-monitored fasting morning blood glucose (finger-stick) levels were measured 3 days weekly in the morning, before caloric intake, and recorded in patient diaries. Values ≥ 270 mg/dL had to be reported directly to the clinic. If a repeat measurement performed within the same week was ≥270 mg/dL, the patient was discontinued from the study and offered rescue medication. Similarly, if 3 or more finger-stick glucose readings within 12 h were <50 mg/dL without a reasonable explanation, the patient was discontinued, and rescue 20 g glucose tablets were administered. Outpatient visits were conducted in Weeks 1, 2, 4, 8, and 12 (final visit). At each visit, concomitant medications were reviewed, weight, vital signs, and blood lipids were measured, clinical laboratory evaluations were performed, and medication was dispensed to last until the next nominal clinic visit. In addition, adverse events were monitored and recorded. At Week 12 subjects, HbA1c was measured, and MRI-PDFF and CAP-Fibroscan were performed. Treatment safety was monitored for an additional 4 weeks after the 12-week treatment period.

### Patients

Main inclusion criteria were patients between the ages of 18 and 70 years and with T2DM, defined by fasting plasma glucose ≥126 mg/dl or 2h postprandial glucose ≥200 mg/dl following 75g OGTT or HbA1c 6.5%–11% or on treatment with at least one and no more than 3 of the following oral antidiabetes medications: metformin, sulfonylurea, DPP-4 inhibitors, oral GLP-1 receptor agonists (Semaglutide), SGLT-2 inhibitor, or thiazolidinediones. Patients also had to have a diagnosis of NAFLD based on a noninvasive determination of hepatic steatosis grade S1, defined as hepatic steatosis>8% by MRI- PDFF and CAP FibroScan ≥ 238 dB/m, fibrosis score 1 ≤ F ≤ 3 and body mass index ≥25. The main exclusion criteria included active liver disease other than MASH, abnormal synthetic liver function, known alcohol and/or drug abuse/dependence, alanine aminotransferase (ALT) or aspartate amino transferase (AST) levels more than the five-time upper limit of normal, weight >120 kg, renal dysfunction, and a clinically significant concomitant disease other than T2DM and MASH. The inclusion and exclusion criteria list can be found in the NIH clinical trials entry.

Follow-up parameters include the following:

a. The primary objective was to evaluate the safety of oral insulin, as assessed by clinical and laboratory parameters.
b. The secondary objectives were to assess whether oral insulin effectively reduces liver fat content, as determined by the median change from baseline at week 12 in FibroScan® fibrosis levels (kPa) and steatosis levels (dB/m), as well as by MRI-PDFF.
c. Patients were followed for a change from baseline at weeks 12 and 16 in HbA1c, lipid profile including total cholesterol, low-density lipoprotein, high-density lipoprotein, and triglycerides, and liver enzymes (ALT and AST).

### Blinding

The study treatment was double-blinded to the subjects, investigators, monitors, and staff responsible for treating and evaluating subjects. The blind was maintained throughout the study.

### Randomization

A computer-generated randomization schedule was used to assign patients to treatment groups. The unblinded investigational site pharmacist or designee followed the randomization schedule to dispense the appropriate study treatment.

### Statistics Analysis

This study was not powered to detect statistically significant differences between treatment groups, and *P* values are not presented for any outcome parameters. Descriptive statistics of change from baseline and value at each time point are presented for each evaluable safety and efficacy parameter.

## Results

Ninety-two patients were screened, 59 failed the screening process, and 32 were randomized to one of the 2 treatment cohorts (placebo: n = 11; ORMD-0801: n = 21). All but 2 ORMD-0801 patients completed the study. In one case, premature discontinuation was due to hypersensitivity, vomiting, and abdominal discomfort considered possibly related to the study drug, and in another, discontinuation was due to an unrelated severe gastrointestinal bleeding event. One subject was randomized but failed the run-in period, was discontinued, and was not included in the safety or efficacy assessments. For another subject, the baseline whole-liver MRI-PDFF score was unavailable; the subject was not included in the primary efficacy analysis.

Patient demographics and baseline characteristics are summarized in Table 1. Most subjects in both cohorts were males (placebo: 54.5%; ORMD-0801: 66.7%), and most of the population in both cohorts was white (∼90%). Mean patient age (56.7 ± 10.4 vs 59.7 ± 7.6 years, respectively) and BMI (32.1 ± 4.5 33.6 ± 5.3 kg/m^2^) were similar in the placebo and active treatment arms. Most patients had a history of vascular disorders (90.9% and 81.0%, respectively). All 11 (100%) patients in the placebo group and 20 (95.2%) patients in the ORMD-0801 group took at least one concomitant medication during the study. Concomitant medications predominantly involved metformin and metformin hydrochloride for the treatment of T2DM and atorvastatin for mixed hyperlipidemia.

**Table 1:** Patient Demographics and Baseline Characteristics

Parameter	Placebo (N = 11)	ORMD-0801 8 mg BID (N = 21)
Sex, n (%)		
Male	6 (54.5)	14 (66.7)
Female	5 (45.5)	7 (33.3)
Race, n (%)		
White	10 (90.9)	19 (90.5)
Black or African American	0	1 (4.8)
Asian	1 (9.1)	0
American Indian or Alaska Native	0	0
Native Hawaiian or other Pacific Islander	0	0
Other	0	1 (4.8)
Not reported	0	0
Ethnicity, n (%)		
Hispanic or Latino	7 (63.6)	11 (52.4)
Not Hispanic or Latino	4 (36.4)	10 (47.6)
Not reported	0	0
Not required	0	0
Age (y)		
Sample size	11	21
Mean	56.73	58.67
Standard deviation	10.355	7.565
Median	58.00	61.00
Min, max	37.0, 70.0	44.0, 69.0
Childbearing status		
Of childbearing potential	0	0
Surgically sterilized	2 (18.2)	4 (19.0)
At least 24 mo postmenopausal	3 (27.3)	3 (14.3)
Multiple items indicated	6 (54.5)	14 (66.7)
Height (cm) at screening		
Sample size	11	21
Mean	164.4	165.0
Standard deviation	13.66	9.63
Median	167.0	164.0
Min, max	143, 187	148, 183
Weight (kg) at screening		
Sample size	11	21
Mean	86.84	91.81
Standard deviation	16.255	18.472
Median	84.10	95.20
Min, max	63.9, 110.4	59.2, 123.0
BMI (kg/m^2^) at screening		
Sample size	11	21
Mean	32.109	33.567
Standard deviation	4.5496	5.2981
Median	32.500	33.700
Min, max	25.10, 39.60	25.20, 44.20
Primary medical history recordings		
Gastrointestinal disorders	5 (45.5)	8 (38.1)
Hepatobiliary disorders	6 (54.5)	16 (76.2)
Metabolism and nutrition disorders	11 (100)	21 (100)
Musculoskeletal and connective tissue disorders	5 (45.5)	4 (19.0)
Nervous system disorders	6 (54.5)	5 (23.8)
Surgical and medical procedures	6 (54.5)	8 (38.1)
Vascular disorders	10 (90.9)	17 (81.0)

Max, maximum; Min, minimum.

### ORMD-0801 Safety

High levels of compliance were documented in both treatment arms, with >90% of capsules administered in both the run-in and treatment phases. No deaths or treatment-related serious or severe adverse events were reported throughout the study period. Two treatment-related events (hypersensitivity/abdominal discomfort and moderately elevated HbA1c, classified as moderately severe) were reported in the ORMD-0801 cohort, the former event leading to study drug withdrawal. One patient in the placebo group discontinued treatment due to moderate hyperglycemia unrelated to the study drug. The second discontinuation was due to a severe event that was unrelated to the drug. It was classified as a severe, treatment-emergent AEs (TEAEs) but not a severe treatment-related event. Overall, there were no significant changes in fasting blood glucose, fasting insulin, HOMA-IR, adiponectin, or safety lab parameters between baseline and Week 12 or Week 16 of the study in either treatment cohort. Similarly, no clinically significant changes in weight, vital signs, ECG, or physical findings were noted between baseline and Week 12 (Table 2).

**Table 2:** Adverse Events Overall Summary

Parameter	Placebo (N = 11)	ORMD-0801 8 mg BID (N = 21)
Number of reported adverse events	11	21
Subjects with at least one: n (%)		
Treatment-emergent adverse event (TEAE)	4 (36.4)	7 (33.3)
Severe TEAE	0 (0.0)	2 (9.5)
Serious TEAE	1 (9.1)	1 (4.8)
Drug-related TEAE	0 (0.0)	2 (9.5)
Drug-related severe TEAE	0 (0.0)	0 (0.0)
Drug-related serious TEAE	0 (0.0)	0 (0.0)
TEAE leading to withdrawal of study drug	1 (9.1)	2 (9.5)
TEAE with outcome of death	0 (0.0)	0 (0.0)

TEAE, Treatment-emergent adverse events.

### Effect of ORMD-0801 on Liver Steatosis

**Figure 1:**
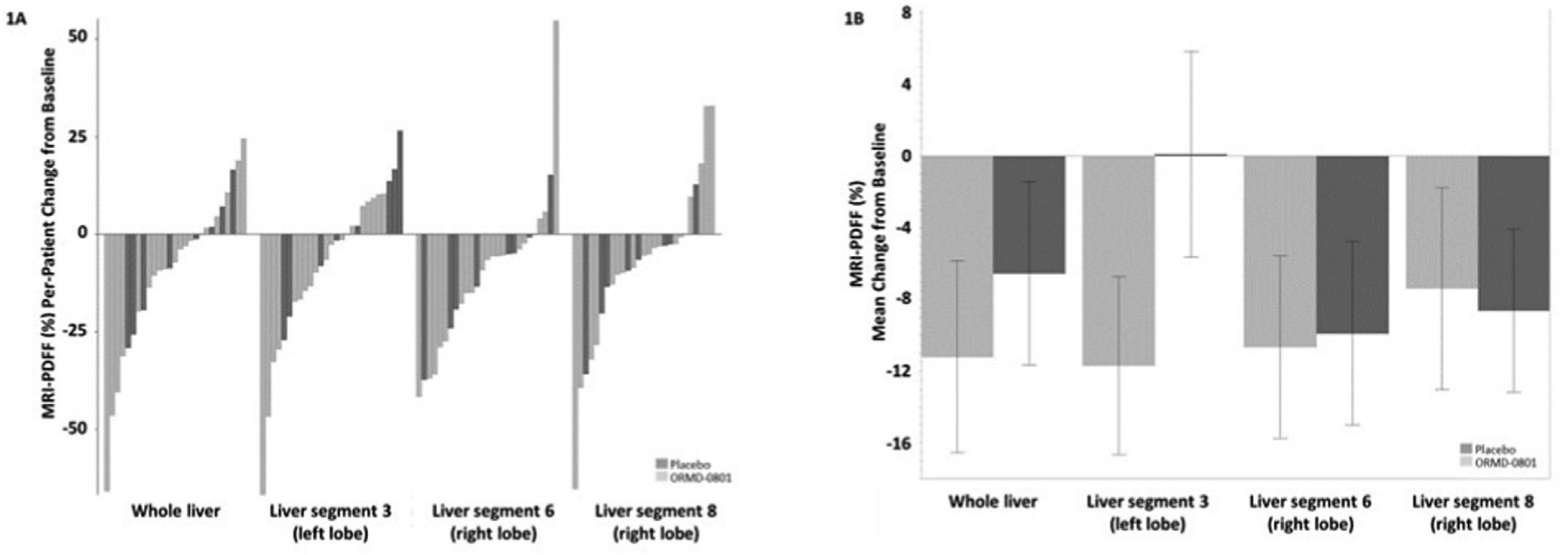
Reduced liver fat content following oral insulin treatment Patients were treated twice daily with either a placebo or an oral 8 mg insulin (ORMD-0801) capsule. Whole-liver and liver segment fat content were evaluated by MRI-proton density fat fractions (MRI-PDFF) at the screening visit and after 12 weeks of treatment. The (A) by-patient percent change (B) and mean percent change from baseline are presented.

**Figure 2:**
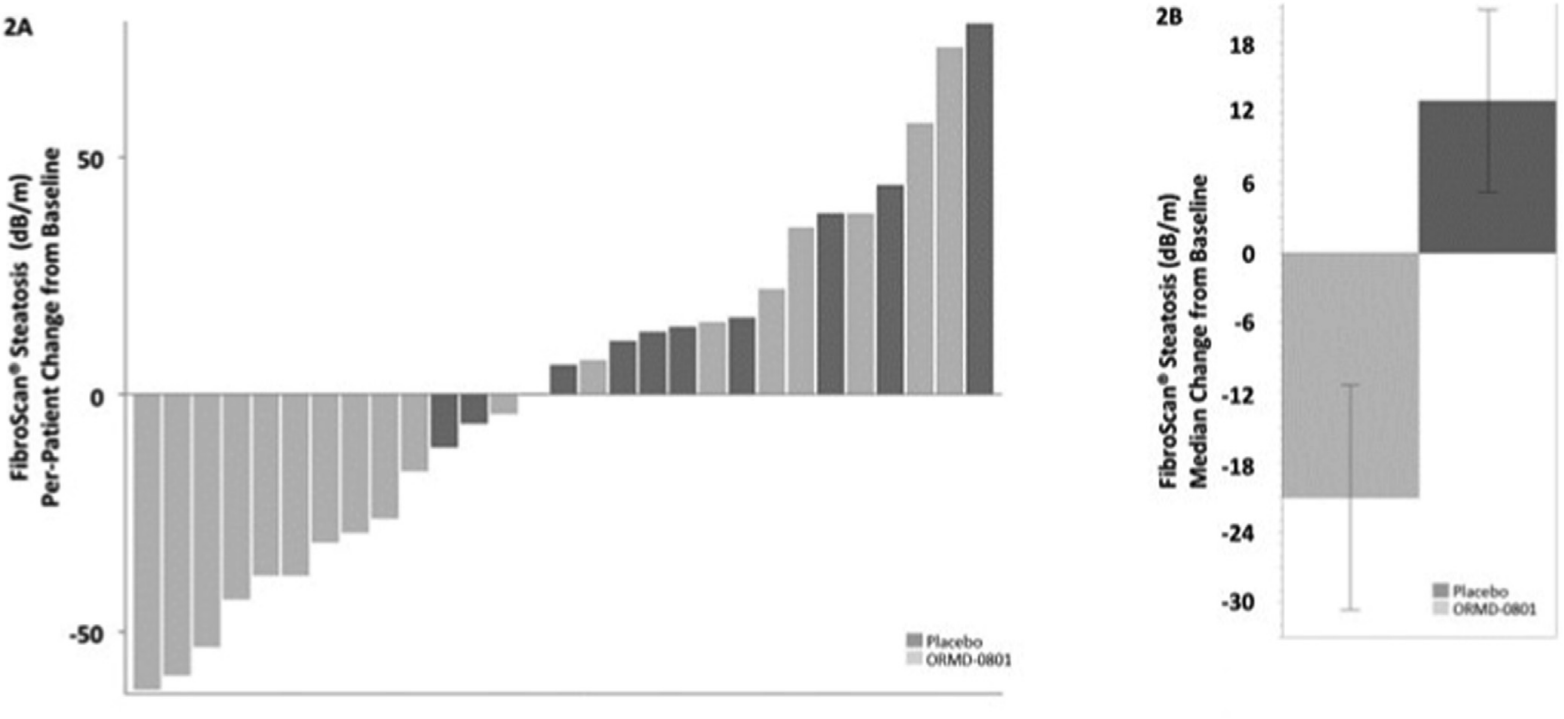
Reduced elastography-measured steatosis following oral insulin treatment Patients were treated twice daily with either a placebo or an oral 8 mg insulin (ORMD-0801) capsule. Liver steatosis was evaluated using a FibroScan® at the screening visit and after 12 weeks of treatment. The (A) by-patient change (B) and median change from baseline are presented.

**Figure 3:**
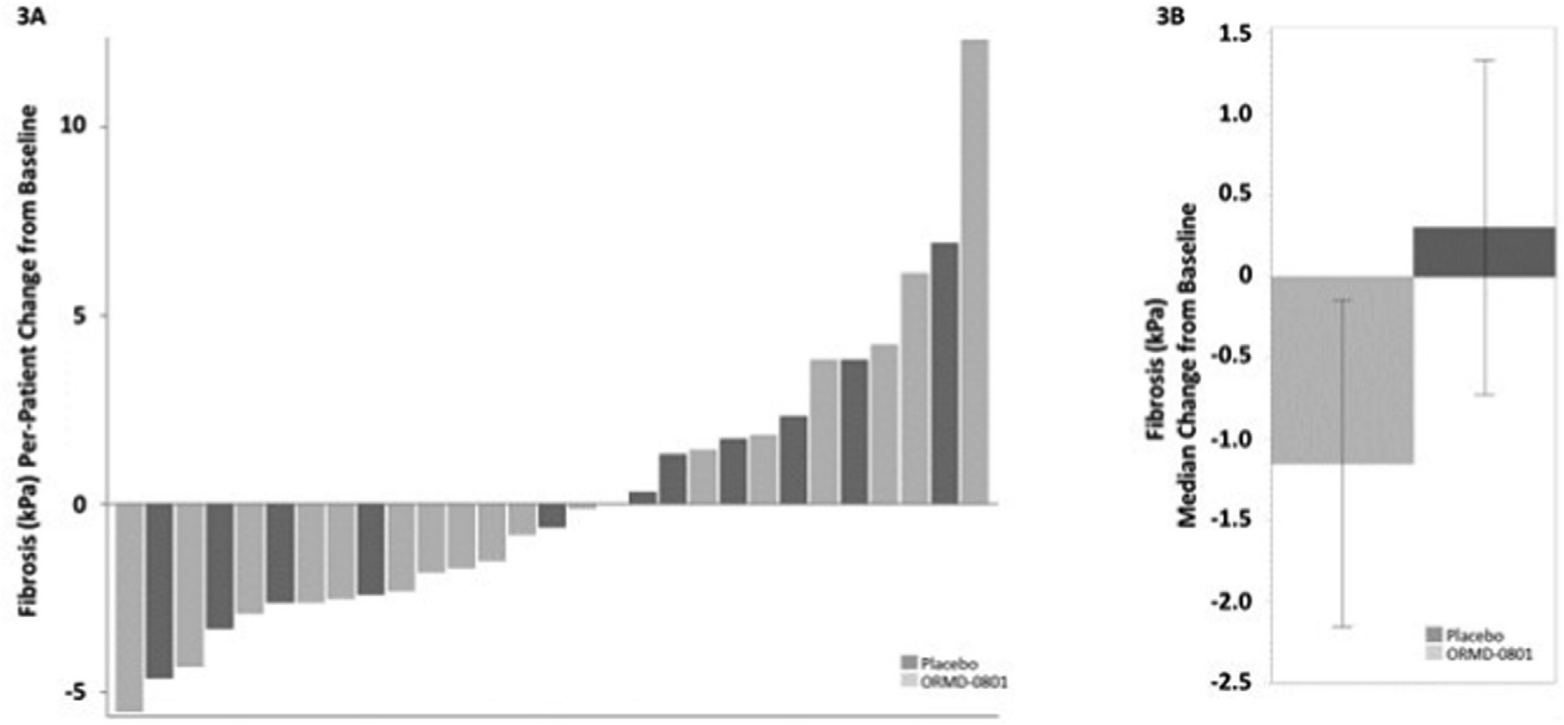
Reduced liver fibrosis following oral insulin treatment Patients were treated twice daily with either a placebo or an oral 8 mg insulin (ORMD-0801) capsule. Liver fibrosis was evaluated using a FibroScan® at the screening visit and after 12 weeks of treatment. The (A) by-patient change (B) and median change from baseline are presented.

Figure 1 shows the by-patient and mean effect of treatment on liver steatosis as measured by MRI-PDFF. After 12 weeks of treatment, mean percent reductions in whole-liver (−11.2% vs −6.5%, respectively) and liver segment 3 (−11.7% vs +0.1%, respectively) fat content was higher in the insulin than in the placebo arm. In line with these findings, individual (Figures 2A and 3A) and median (Figures 2B and 3B) changes in FibroScan® steatosis and fibrosis were more prominent in the active treatment than in the placebo arm. Patients receiving insulin showed a median −1.1 kPa and −21.0 dB/m reduction from baseline fibrosis and steatosis levels, respectively, while placebo-treated patients showed median increases of 0.3 kPa and 13.0 dB/m, respectively.

A reduction from baseline steatosis by week 12 was documented for 55.0% of the patients receiving oral insulin compared to 1.2% in the placebo arm. In addition, compared to the placebo arm, a more significant percentage of patients receiving active treatment showed a ≥5% reduction in whole-liver fat content by week 12 (36.4% vs 50%, respectively).

### Effect of ORMD-0801 on HbA1c, Lipid Profile, and Liver Enzymes

**Figure 4:**
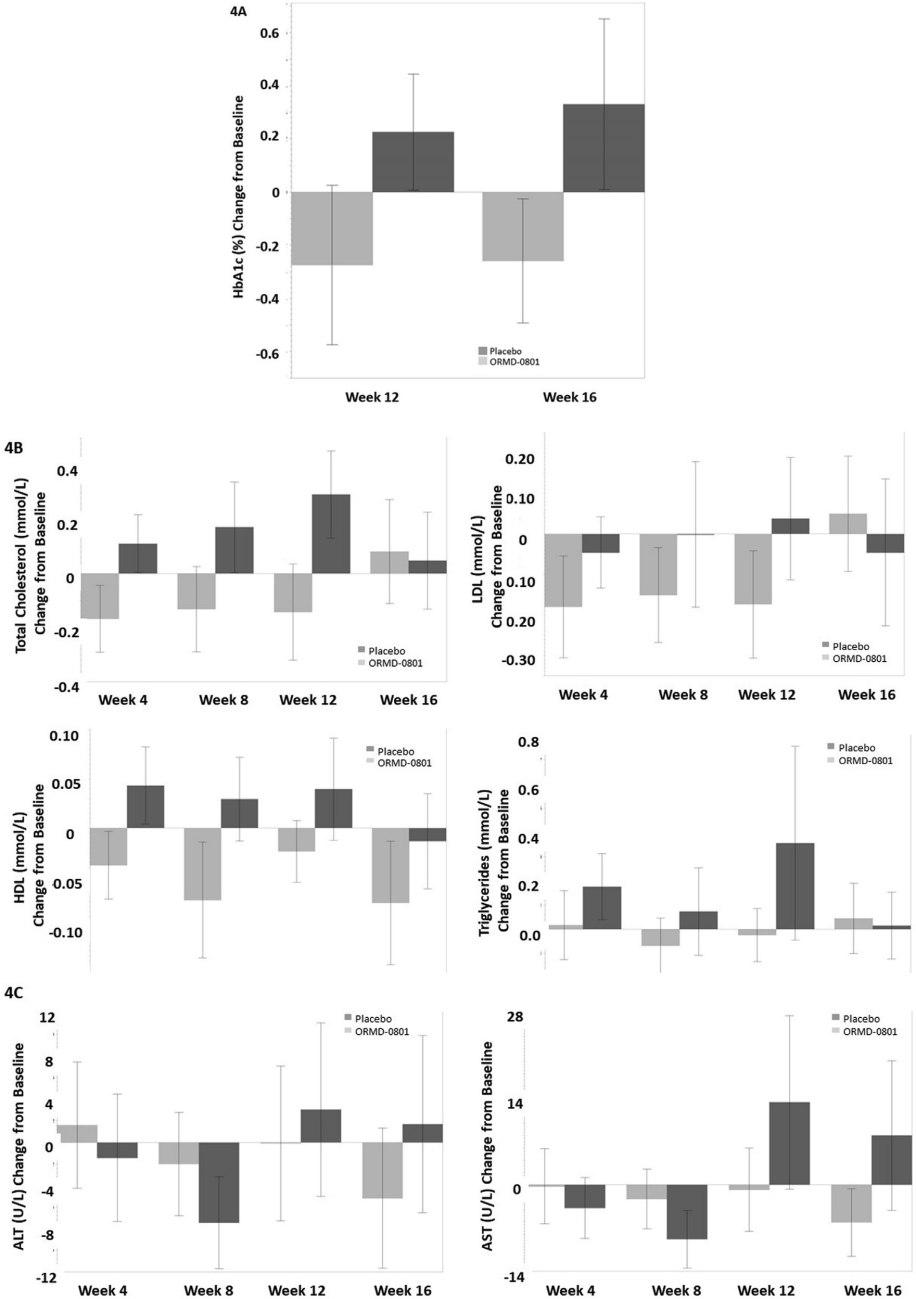
Reduced insulin resistance, blood lipids and liver enzyme levels following oral insulin treatment Patients were treated twice daily with either a placebo or an oral 8 mg insulin (ORMD-0801) capsule. Blood samples were collected at baseline, after 12 weeks of treatment and 4 weeks after treatment termination (week 16). Mean change from baseline (A) hemoglobin A1c (HbA1c), (B) total cholesterol, high-density and low-density lipoproteins and triglycerides, and (C) aspartate amino transferase (AST) and alanine aminotransferase (ALT) levels are presented.

At Week 12, oral insulin was associated with a mean of 0.27% reduction, and placebo with a mean of 0.23% increase from baseline HbA1c levels (Figure 4A). Mean total cholesterol, high-density lipoprotein, low-density lipoprotein, and triglyceride levels were consistently lower than baseline levels in the active arm and higher than in the placebo arm (Figure 4B). Mean percent changes from baseline ALT and AST levels were −1.1% and −0.8% in the oral insulin and 3.0% and 13.4% in the placebo arm (Figure 4C).

## Discussion

The data of the present randomized, placebo-controlled feasibility trial support the high safety profile of ORMD-0801 in patients with MASH and T2DM, which was also documented in larger cohorts of patients with T2DM.^17^^,^^18^

The results may suggest a positive effect of ORMD-0801 on insulin resistance, an essential variable in the mechanism of the disease.^19^ ORMD-0801 had a beneficial effect on steatosis, as shown by MRI and Fibroscan, and fibrosis, shown by Fibroscan. The percentage of patients with reduced liver fat was higher in the treated group than in the placebo. The noted effects on segment 3 suggest fat redistribution in the liver, and a more extended treatment period may have shown a profound effect on the whole liver. Improved hepatic sensitivity may be associated with the noted positive effect on steatosis and fibrosis. The ORMD-0801 formulation has physiologic advantages since it recapitulates the natural route of insulin secretion and absorption through the portal vein and targets the liver directly.^20^

Insulin resistance plays a role in the pathogenesis of MASH.^4^^,^^8^^,^^21^^,^^22^ It is associated with the activation of multiple pathways that underlie inflammatory processes and fibrosis development in these patients. The mechanisms driving these processes involve hepatic fat accumulation, alterations in energy metabolism, genetic factors, and inflammatory signals from immune cells. Hepatic stellate cells, the primary cell type responsible for extracellular matrix production, are activated by insulin resistance.^23^^,^^24^ Dysfunctional insulin-resistant adipose tissue underlies the development of advanced fibrosis in T2DM beyond the BMI or steatosis.^25^ Subjects with hepatic steatosis alongside insulin resistance had higher odds of fibrosis, supporting the importance of the metabolic aspects of the disease and specifically of insulin resistance on fibrosis risk.^22^ The metabolic score for insulin resistance is inversely related to advanced fibrosis in patients with NAFLD.^26^

Safety is a significant concern when targeting patients with MASH, which requires many years of therapy.^27^^,^^28^ The high safety profile of ORMD-0801 and the lack of hypoglycemic effect support its potential use for long periods in this patient population. Based on the current data, the 0% vs 9.5% severe events rate in placebo vs treated seems clinically insignificant.

The multiple mechanisms that underlie the development of MASH, comprising both metabolic derangement activation of inflammatory, microbiome-related, and pro-fibrotic pathways, mandate combination therapies for achieving clinically meaningful results.^12^^,^^29^^,^^30^ The data presented suggests that the availability of a safe, orally administered product that targets insulin resistance may enable the use of a combination of ORMD-0801 with other antifibrotic and anti-inflammatory agents currently being developed for MASH.^12^

The use of oral insulin formulation may also affect the immune system by targeting the gut immune system in a way that alleviates systemic inflammation.^31^, ^32^, ^33^ Oral immunotherapy was proposed to alleviate liver damage in MASH.^31^^,^^33^, ^34^, ^35^, ^36^ The effect of ORMD-0801 on MASH-associated inflammatory pathways remains to be determined in future trials.

Insulin resistance was described in lean and overweight patients with MASH.^37^^,^^38^ In the present study, there was no change in body weight, which is vital as it supports a direct effect of the drug on the disease. It also suggests that ORMD-0801 can be used in lean patients with MASH who have insulin resistance.

The limitations of the present study are its lack of power to achieve statistically significant results and the relatively short follow-up period. Nevertheless, the data showing positive trends in all parameters tested are encouraging and suggest a biological effect of oral insulin in MASH. The fact that the improved parameters in the liver continued during the follow-up period further supports the drug's biological effect.

## Conclusion

The results of this feasibility study demonstrated the safety and potential therapeutic effect of orally delivered insulin formulations on liver fibrosis, fat accumulation, and inflammatory processes. Large controlled trials for extended periods are ongoing to determine and better characterize the beneficial effects of ORMD-0801 in patients with MASH.
